# Synthesis, characterization and heavy metal removal efficiency of nickel ferrite nanoparticles (NFN’s)

**DOI:** 10.1038/s41598-021-83363-1

**Published:** 2021-02-15

**Authors:** Waheed Ali Khoso, Noor Haleem, Muhammad Anwar Baig, Yousuf Jamal

**Affiliations:** 1 Institute of Environmental Sciences and Engineering (IESE), School of Civil and Environmental Engineering (SCEE), National University of Sciences and Technology, Islamabad, 44000 Pakistan; 2 Institute of Chemical Engineering & Technology, University of Punjab, Lahore, 54590 Pakistan

**Keywords:** Biotechnology, Biogeochemistry, Environmental sciences, Chemistry, Materials science, Nanoscience and technology

## Abstract

The heavy metals, such as Cr(VI), Pb(II) and Cd(II), in aqueous solutions are toxic even at trace levels and have caused adverse health impacts on human beings. Hence the removal of these heavy metals from the aqueous environment is important to protect biodiversity, hydrosphere ecosystems, and human beings. In this study, magnetic Nickel-Ferrite Nanoparticles (NFNs) were synthesized by co-precipitation method and characterized using X-Ray Diffraction (XRD), Energy Dispersive Spectroscopy (EDS) and Field Emission Scanning Electronic Microscopy (FE-SEM) techniques in order to confirm the crystalline structure, composition and morphology of the NFN’s, these were then used as adsorbent for the removal of Cr(VI), Pb(II) and Cd(II) from wastewater. The adsorption parameters under study were pH, dose and contact time. The values for optimum removal through batch-adsorption were investigated at different parameters (pH 3–7, dose: 10, 20, 30, 40 and 50 mg and contact time: 30, 60, 90, and 120 min). Removal efficiencies of Cr(VI), Pb(II) and Cd(II) were obtained 89%, 79% and 87% respectively under optimal conditions. It was found that the kinetics followed the pseudo second order model for the removal of heavy metals using Nickel ferrite nanoparticles.

## Introduction

Heavy metals ions deteriorate the groundwater quality to the level of being hazardous^1^ and non-biodegradable nature to meet the local environmental standards before it is recycled or returned to major resources of water^2^. Heavy metals cause serious problem due to their toxic effect on humans and the environment^3^. Unlike organic pollutants because they are bio-degradable but heavy metal do not degrade into harmless end products^4,5^. Industries including; mining, paints and pigment, fertilizer, textile, metal plating, batteries and electronic waste are the major sources that discharge heavy metals directly or indirectly into the environment^1,6^.

The United States Environmental Protection Agency (US EPA) has established maximum contaminant level (MCL) for different heavy metals beyond which these are considered to be toxic for human consumption^7^ and the values for Chromium^8^, Lead and Cadmium are 0.05, 0.006 and 0.01 mg/L respectively^9–11^. Even at low concentrations their ions have been found toxic to aquatic flora and fauna. Since last few decades, various techniques such as chemical precipitation, membrane separation, electrolytic separation, ion exchange and adsorption have been used to reduce these toxic metals from aqueous medium^12^. Especially the role of nanomaterials is emerging as an effective adsorbent for wastewater treatment^13,14^.

Adsorption technique has been widely used for the removal of heavy metals. Several researches have shown that nanomaterials are effective sorbents for the removal of heavy metal ions from wastewater^2,15^. Adsorption with nanomaterials is a finest method for heavy metal removal based on the physical interaction between metal ions and sorbents^7^. Adsorbents based on nickel ferrite nanoparticles (NFNs) is seen as potential for heavy metal removal in view of its low cost, high efficiency^7^, and simplicity of operation for removing trace levels of heavy metal ions^16,17^. Furthermore, the sludge produced from the adsorption process is also considered to be simpler for disposal than that from other processes^18,19^.

Due to their small size and large surface area, nanomaterials have strong adsorption capacities and reactivity^20^. Moreover, the mobility of nanomaterials in solution is high. Heavy metals, organic pollutants, inorganic anions and bacteria have been reported to be successfully removed by various kinds of nanomaterials^21^. Recently, research on Nickel Ferrite Nanoparticles (NFNs) have attained considerable attention^22^ because of the difference of their physical and chemical properties from those of the free atoms or molecules^8,16,23^ as well as from the properties of the bulk solids and have wide range of potential applications. Due to its typical ferromagnetic properties, low conductivity, lower eddy current losses and high electrochemical stability. Nickel ferrite are reported as one of the multifaceted significantly soft ferrite materials. The site of the divalent cations (Ni^2+^) is homologous to the magnetic properties in the crystal structure of the nickel ferrite. Moreover, nickel ferrite shows super-paramagnetic property and it has diverse applications such as gas-sensor, catalyst, magnetic fluids and magnetic storage systems^24^.

For synthesis of various metal-oxide nanoparticles, several chemical methods have been reported in literature such as hydrothermal, reverse micelle, solvothermal, sol–gel, polymeric precursor and co-precipitation^19^. This paper aims to report the synthesis of Nickel Ferrite Nanoparticles (NiFe_2_O_4_) by using co-precipitation method. The potential use of these nanoparticles as adsorbent^22^ for removal of heavy metals (Cr(VI), Pb(II) and Cd(II)) from synthetic wastewater was investigated particularly^25^. For this purpose, the influence of pH, contact time and initial does on the sorption capacity of NFNs has been studied. We have also investigated the different effect of pH on adsorption of Cr (VI) and Pb and Cd. Pseudo second order kinetic model were used for the surface adsorption to explain the adsorption kinetics most effectively that involves chemisorption, where the removal from a solution is due to physicochemical interactions between the two phases^26^.

## Materials and methods

### Materials

#### Material for synthesis of NFNs

Following chemicals were used for the synthesis of nickel-ferrite nanoparticles NFN’s.

- Nickel nitrate hexahydrate (Ni (NO3)_2_·6H_2_O)
- Ferric chloride (FeCl_3_)
- Hydrochloric acid (HCl)
- Sodium hydroxide (NaOH)
- Potassium dichromate (K_2_Cr_2_O_7_)
- 1, 5-Diphenylcarbohydrazide (C_13_H_14_N_4_O)
- Ethanol (C_2_H_5_OH)
- Acetone (C_3_H_6_O)
- Sulphuric acid (H_2_SO_4_)
- Oleic acid (C_18_H_34_O_2)_

All the chemicals used in synthesis of NFN’s were of analytical grade.

### Preparation of nickel-ferrite nanoparticles (NFNs)

Magnetic NFN’s were prepared by the co-precipitation method in which nickel nitrate and ferric chloride salts are mixed in 1:2 molar ratios before being extracted through precipitation at high pH.

#### Nickel nitrate hexahydrate (Ni (NO_3_)_2_·6H_2_O) solution

In order to prepare 0.2 M solution, 2.909 g of nickel nitrate hexahydrate was dissolved in 50 ml distillated water to make 50 ml of 0.2 M solution and 5.406 g of Ferric chloride salt (FeCl_3_) was dissolved in distillated water to make 50 ml of 0.4 M solution of Ferric Chloride (FeCl_3_). Both solutions were mixed properly on magnetic stirrer at 200 rpm homogenously at 80 °C for 30 min, then placed outside room temperature. Sodium hydroxide (12 g) was mixed in 100 ml distilled water to make 100 ml of 2.0 M solution of mineralized sodium hydroxide (NaOH), which was then added drop wise using syringe as precipitating agent until the solution gained required pH of 8–9, while constantly stirred at 400 rpm and maintained at a temperature of 70 ± 5 °C for 2 h till brown precipitates were formed. After that, the stirrer was switched off and the magnetic particles settled gradually. The brown precipitate product was cooled at room temperature and the precipitated solution was washed several times with distilled water and neutralized with ethanol (C_2_H_5_OH) to eliminate unwanted impurities from the sample by centrifugation at 4000 rpm. The obtained substance was placed in an oven at 70 °C for 24 h, and after drying, the dried nano powder was ground and calcined at 350–550 °C for 3 h in a muffle furnace to obtain pure NiFe_2_O_4_, which was then crushed by pestle to obtain the final product in a fine powder form.

### Characterization of nanoparticles

For the purpose of characterization following three techniques were employed to verify size of synthesized NFNs.

#### X-Ray diffraction (XRD)

X-Ray diffraction (XRD, JEOL JDX-II) was used to find out the crystalline phase and size of nanoparticles using the Shimadzu-7000 X-ray diffractometer with monochromatized CuKa radiation^27^. The measurement was performed in the range from 20° to 80°.

#### Field emission scanning electron microscope (FE-SEM)

The topography and morphology of the Nickel-ferrite nanoparticles (NFN’s) was carried out using scanning electron microscope (JEOL JSM-6490) at high magnifications. To avoid the charging effect samples were coated with gold for SEM analysis.

#### Energy dispersive spectroscopy (EDS)

Energy-dispersive spectroscopy (EDS, JEOL JSM 6490A) technique was used to determine the elemental composition of selected area on the surface of sample.

### Determination of heavy metal concentrations

UV/Vis Spectrophotometer with quartz cell was used for the detection and quantization limits of Cr(VI) by measuring absorbance values at the wavelength of maximum absorbance at 540 nm using the T60 PG instrument, Calibration curves were created using reagent manufacturer for simple quantitative determinations.

#### Preparation of stock solutions for adsorption study

The Cr (VI) stock solution of 5 mg/L was prepared by dissolving 0.1414 g of potassium dichromate (K_2_Cr_2_O_7_) in distilled water and diluted to 100 mL; 1.00 mL = 500 μg of Cr. This was further diluted to create another standard stock solution of 0.05 mg/L (100 mL = 0.05 mg/L of chromium); this stock solution was used for adsorption experiments and was then analyzed using UV/Vis spectrophotometer. Following the same above procedure of chromium solution for preparation of lead stock solution by dissolving 0.15985 g of Lead Nitrate [Pb (NO_3_)] in distilled water and diluted to 100 mL to prepare; (1.00 mL = 5 ppm of Pb). The solutions with 5 mg/L concentrations of the lead was prepared by dilution of stock solution with ultrapure water, producing a 5 mg/L lead standard stock solution of 1000 ml. This was further diluted to create another standard stock solution of 0.5 mg/L (10 mL = 0.5 ppm of lead); this stock solution was used for adsorption experiments and analyzed in Atomic Adsorption Spectrophotometer (AAS).

Preparation of cadmium stock solution by dissolving 0.2744 g of Lead Nitrate (Pb (NO_3_)) in distilled water and diluted to 100 mL was achieved. to prepare. The solution with 5 ppm concentrations of cadmium was prepared by dilution of the stock solution with ultrapure water, producing a 5 mg/L cadmium standard stock solution of 1000 ml. This was further diluted to create another standard stock solution of 0.5 mg/L; this stock solution was used for adsorption experiments and analyzed in Atomic Adsorption Spectrophotometer (AAS).

### Preparation of calibration and standard curve for chromium detection

Standard method (3500-CR) was used to determine Cr (VI) in prepared solution using UV/Visible spectrophotometer. For this reason, UV–Vis spectrum of a dye Diphenyl carbazide (DPC) was recorded for determining Cr (VI) in prepared solution to validate the wavelength of maximum absorbance at 540 nm, which follows the standard method. The calibration curve was developed at λ-max 540 nm using serial dilutions starting from 0.5 to 5 µg/L five samples of different volume of prepared solution as shown in Fig. 2.

Prepared five samples S_1,_ S_2,_ S_3,_ S_4_ and S_5_ of different volume 1, 2, 5, 7 and 10 mL respectively of a 5 µg/L Cr and pH were adjusted to 2.0 ± 5. Transfer of solution to 100 mL volumetric flask and were adding three drops of Phosphoric acid (H_3_PO_4_) plus adding of 2 mL of prepared 1, 5-diphhenylcarbazide solution and mixed, let stand 5 to 10 min for full color development. Transfer an appropriate portion to absorption cell and measure its absorbance at 540 nm wavelength in a spectrophotometer.

### Adsorption experiments batch study

The solutions having 5 mg/L concentrations of Cr(VI) was prepared by dilution of the stock solution using ultrapure water. Adsorption studies were carried out by mixing 10, 20, 30, 40 and 50 mg of Ni-Fe_2_O_4_ adsorbent with 50 mL Cr(VI) solutions of 5 mg/L. The solution was mixed over magnetic stirrer at 200 rpm with different pH and contact time, 3, 4, 5, 6 and 7 and 30, 60, 90, and 120 min respectively as shown in Table 1. The pH values of Cr (VI) solutions were adjusted by using 1 mol/L HCl and 1 mol/L NaOH as reagents.

**Table 1:** Experimental set up for metals adsorption from synthetic wastewater.

Dose/pH	pH levels maintained (3, 4, 5, 6 and 7)			
Adsorbent dose (mg) applied at each pH level	10, 20, 30, 40, 50	10, 20, 30, 40, 50	10, 20, 30, 40, 50	10, 20, 30, 40, 50
Contact time (min)	30	60	90	120

Adsorption was measured by using measuring 50 mL of the synthetic wastewater sample and poured into a 100 mL Backers. 10, 20, 30, 40 and 50 mg of the synthesized nickel nanoparticles were added to different backers containing 50 mL of synthetic wastewater. The backers containing the adsorbent and the synthetic wastewater were placed on a magnetic stirrer at 200 rpm at room temperature for a period of 120 min to ensure equilibrium. The suspension was filtered using 0.22-micron filter paper. UV/Vis spectrophotometer and Atomic adsorption spectro-photometer (AAS) were used to analyze the concentration of the different metal ions present in the filtrate. The amount of metal ions adsorbed by the adsorbent was evaluated and following formula was used to calculate the percentage removal.

1 $${\text{E }} = {\text{ C}}_{{\text{o}}} {-}{\text{ C}}_{{\text{e}}} /{\text{ C}}_{{\text{o}}} \times { 1}00$$

where (E %) is the ratio of difference in metal concentration before and after adsorption.

## Results and discussion

### Characterization of nanoparticles

**Figure 1:**
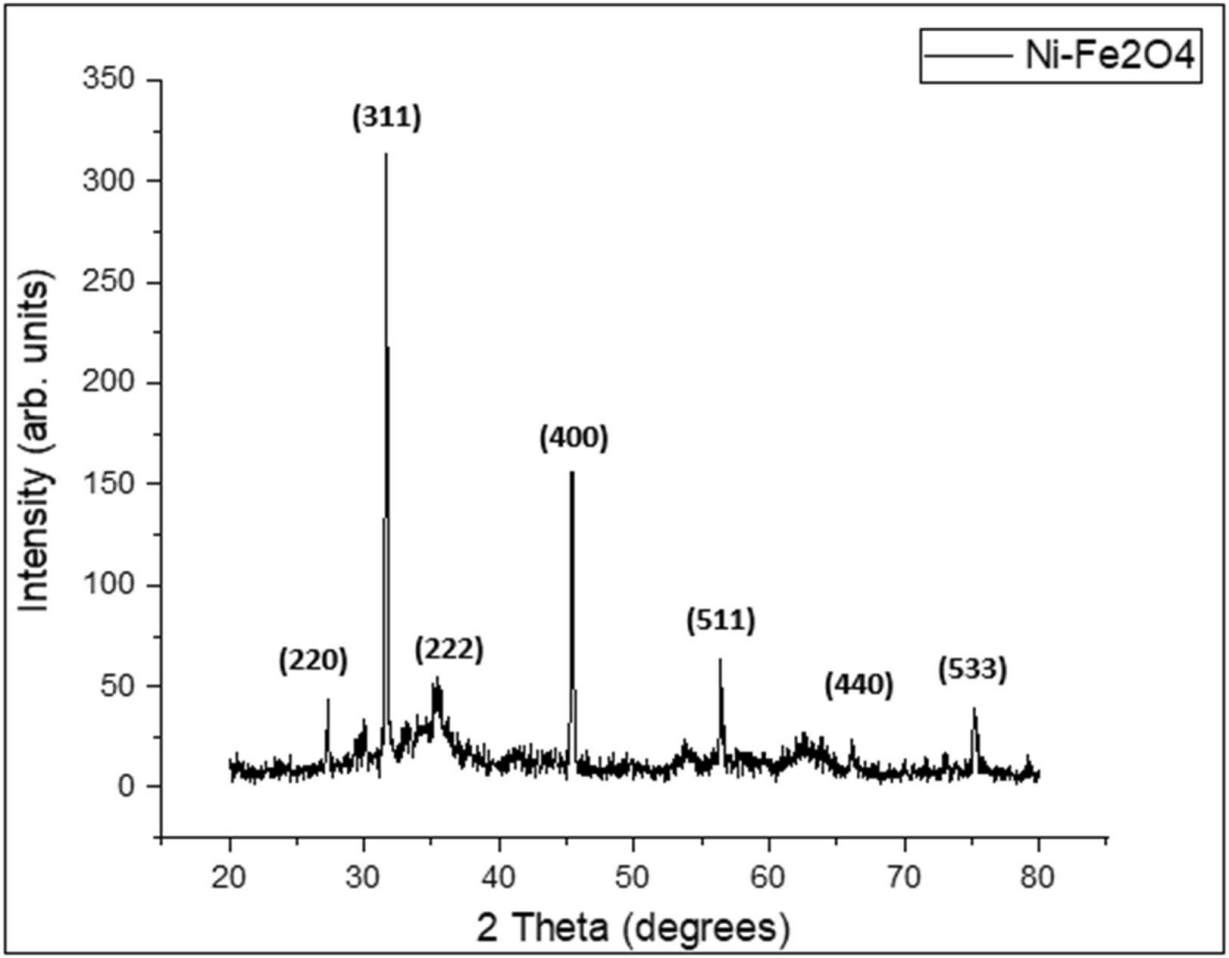
XRD pattern of synthesized nickel ferrite nanoparticles.

The X-ray diffraction patterns of the synthesized nickel ferrite nanoparticles (NFN’s) are shown in Fig. 1. XRD spectrum made it clear that sample has single spinal shape with good crystallinity. The NFN’s contained no impurity peaks within the limit of X-ray detection. The significant peak broadening indicates the ultra-fine nature of the sample. The average crystalline diameter (D) was calculated by Scherer’s equation (D = 0.9λ/βcosθ) as 30.254 nm.

The peaks pattern shown in Fig. 1 of Nickel ferrite are intense and sharp, representing good crystallinity of the prepared sample. The peaks were found to be at 2θ values of 29.84807°, 31.63629°, 35.27°, 45.37836° and 56.43597° corresponded to the crystal planes of (220), (311), (222), (400), (511), (440) and (533) in the pattern of Nickel ferrite, this proved the formation of NFN’s spinel phase. No other peaks are found that indicating the synthesized sample consists of high purity NiFe_2_O_4_. By applying the Scherer’s formula D = 0.9λ/βcos θ, size of the crystallite was calculated. Where D is the crystallite size, β is the line broadening at the full width at half maximum (FWHM) of the most intense peak (311), K is Scherrer constant, θ is the Bragg’s angle and λ is the X-ray wavelength. The average crystallite size of nickel ferrite nanoparticles was estimated to be 30.254 nm.

**Figure 2:**
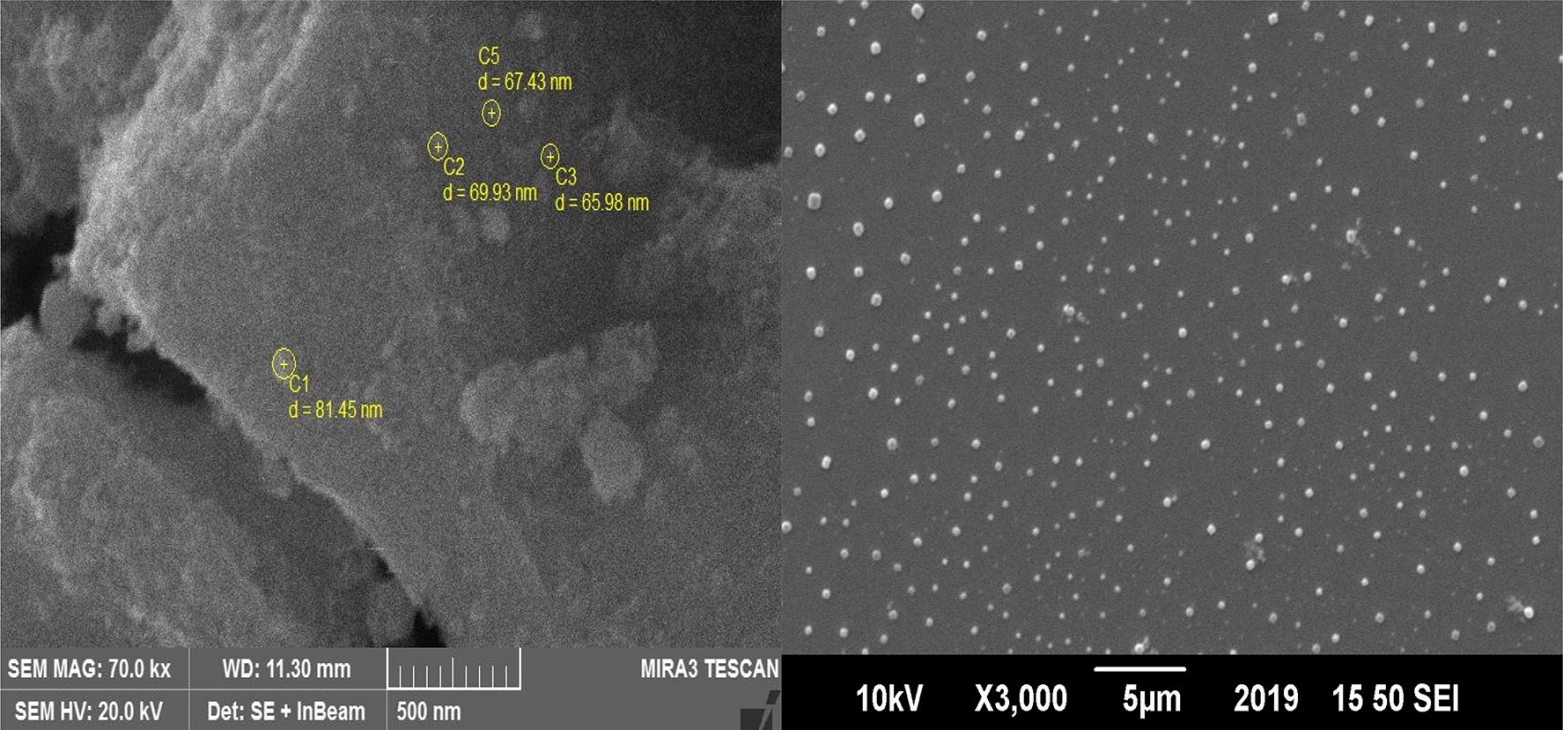
SEM images of NiFe_2_O_4_ nanoparticles.

Figure 2 depicts the field emission scanning electron micro graph (FE-SEM) of magnetic NiFe_2_O_4._ nanoparticles. The SEM image shows that the nanoparticles have sizes of less than 100 nm and they are dense and frequently distributed with-in the whole area. Moreover, aggregation of small particles was also observed. Thus, it has been confirmed and made clear by SEM analysis that spherical shape materials are formed as a result of tiny particle aggregation. The presence of pore free crystallites on the surface is due to agglomeration of small particles as the nanomaterials possess high surface energies. The average size range of nickel ferrite was found to be 30.254 nm.

Energy dispersive X-ray spectroscopy (EDS) is the technique use for the determinations of elemental composition or chemical characterization of sample. Table 2 shows relative elemental mass of different elements such as nickel, iron, and oxygen present in the NFN’s sample. While there is no any alien element or impurity is present in the prepared sample.

**Table 2:** Relative elemental mass of synthesized NFN’s.

Element	Weight %	Atomic %
O K	25.79	44.79
Na K	18.22	22.03
Cl K	18.96	14.86
Fe K	32.69	16.27
Ni K	4.34	2.06

**Figure 3:**
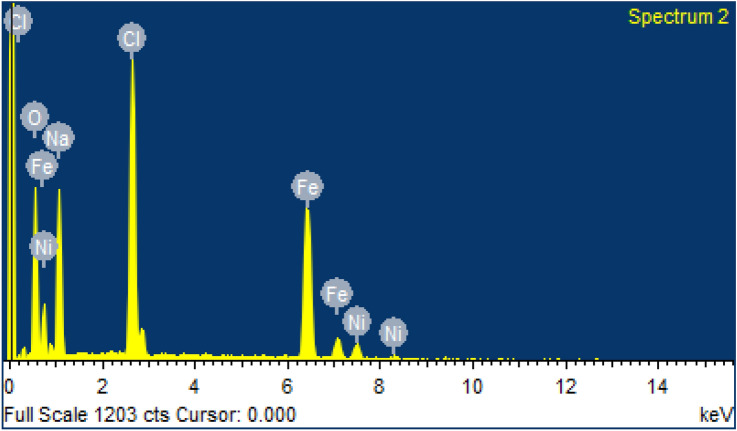
EDS—elemental composition of NiFe_2_O_4_ nanoparticles.

The EDS spectrum is shown in Fig. 3. Peaks corresponding to chlorine, nickel, iron and oxygen were found in the spectrum. A sharp peak of chlorine was observed due to its reactivity. Chlorine tends to react with compounds including metal–metal, metal–hydrogen or metal–carbon bonds to form metal–chlorine bonds as reported ^28^.

### Preparation of calibration curves for analysis

**Figure 4:**
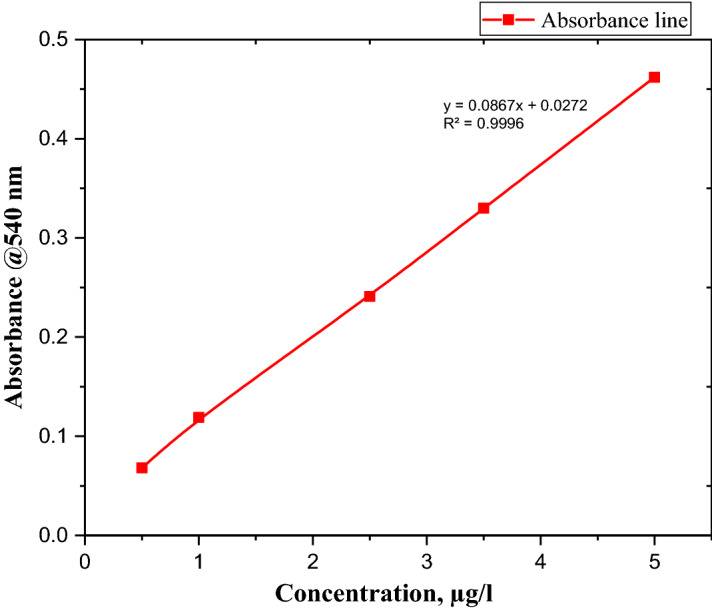
Standard calibration curve spiked with different volumes at λ-max 540 nm.

A calibration curve was developed at λ-max 540 nm using serial dilutions starting from 0.5 to 5 µg/L of solution as shown in Fig. 4 and summarized in Table 3. The calibration curve of Cr (VI) was prepared from five different concentrations 0.5 µg/L, 1 µg/L, 2.5 µg/L, 3.5 µg/L and 5 µg/L standards. The linear calibration curve with the equation absorbance = 0.0867x + 0.0272, where absorbance units are in milli absorbance and concentration is measured in µg/L, gave a correlation coefficient R2 ≥ 0.9996.

**Table 3:** Absorbance of five standard synthesized samples of a 5 µg/L Cr(VI) at 540 nm.

Samples run	Volume (mL)	Absorbance (540 nm)	Concentration (µg/L)
S_1_	1	0.068	0.5
S_2_	2	0.119	1
S_3_	5	0.241	2.5
S_4_	7	0.33	3.5
S_5_	10	0.462	5

A calibration equation (*y* = 0.0867x + 0.0272, *R*2 = 0.9996, where y is absorbance and x is concentration in ppm or µg/l) derived from a calibration curve was plotted from standards (0.5 µg/L, 1 µg/L, 2.5 µg/L, 3.5 µg/L, and 5 µg/L) for the quantization of Cr (VI) in wastewater samples. However, due to the low sensitivity to low Cr (VI) concentrations and low detection limits of Cr(VI) in synthesized samples, light pink color developed with 1,5-diphenylcarbazide. Synthesized samples were spiked with 1 mL, 2 mL, 5 mL, 7 mL and 10 mL of a 5 µg/L Cr(VI) standard. Figures 4 show the spiked curves that were used for the quantization of Cr(VI) in wastewater samples.

### Adsorption batch study

#### Effect of contact time for removal of heavy metals

**Figure 5:**
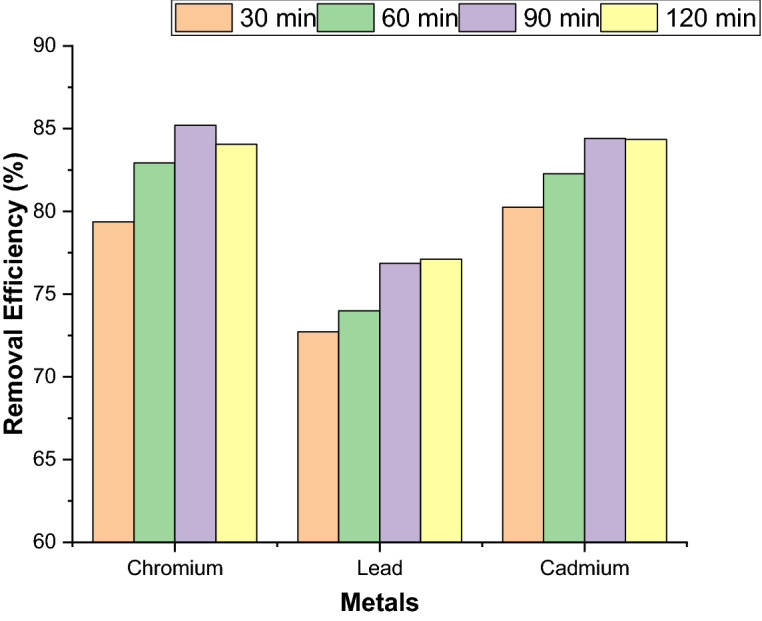
Effect of contact time for removal of Cr(VI), Pb(II), and Cd(II) by adsorption.

The effect of contact time on the removal efficiency of Cr(VI), Pb(II) and Cd(II) ions using synthesized nickel ferrite nanoparticles (NFN’s) were studied at room temperature and the obtained results are shown in Fig. 5.

It was observed that, the removal of metal ions increases with increase in contact time and attained equilibrium level at 90 min, after which further increase in time did not bring any further improvement for the removal of metal ions, rather resulted in desorption of metal ions from the adsorbent surface. Nickel ferrite nanoparticles shows maximum removal efficiency of 85.21% for Cr(VI) ions in 90 min, 77.41% for Pb(II) ions in 120 min, and 84.45% for Cd(II) ions in 90 min applied constant (e.g. 10 mg) of adsorbent dosage. The results shown that the different metal ions attained equilibrium at different times and the higher removal efficiency is for the removal of Cd(II) and lower removal efficiency is for the removal of Pb(II).

#### Effect of pH for removal of heavy metals

**Figure 6:**
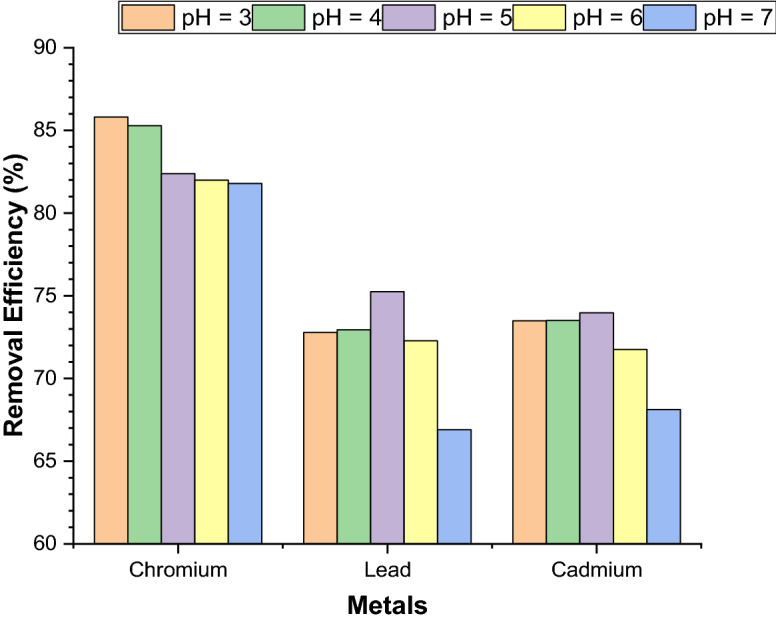
Effect of pH for removal of Cr(VI), Pb(II), and Cd(II) by adsorption.

The effect of pH was studied from a range of 3 to 7 under the precise conditions (at optimum contact time 30 to 120 min @ 200 rpm shaking speed, with 10 mg of the adsorbent used at room temperature). In all experiment the NFN’s dose (10 mg) was kept constant to assessed effect of pH on adsorption of metal ions using nickel ferrite nanoparticles (NFN’s) is shown in Fig. 6.

The effects of solution pH on the adsorption of Cr(VI), Pb(II) and Cd(II) onto NFNs were studied by varying the aqueous solution pH. It was observed that with increased in the pH from 3 to 7 of the synthetic wastewaters, the removal efficiencies of Cr(VI) decreased at rate as pH increased and maximum removal efficiency was obtained at pH 3 as shown in the Fig. 6. Hence, the removal efficiency of chromium gradually decreased as pH increased. The maximum removal efficiency of Lead and Cadmium was found at pH 5 as shown in the Fig. 6. Nickel ferrite nanoparticles (NFN’s) shown maximum removal efficiency of 85.8% for Cr(VI) ions at pH 3, 75.25% for Pb(II) ions at pH 5, 73.97.41% for Cd(II) ions at pH 5. It was confirmed that the adsorption of Pb(II) and Cd(II) increased with the increase of pH value because, at lower pH, more hydrogen ions (H^+^) were contained in the system and the high concentration was competitive with both metal ions which causes low adsorption. As the solution pH increased, the adsorption of metal ions on the NFNs increased due to the decreasing H^+^ ions. These ions acted as a competitor to the positive metal ions for adsorption sites on the surface of the nanocomposite.

##### Effect of absorbent dosage for removal of heavy metals

**Figure 7:**
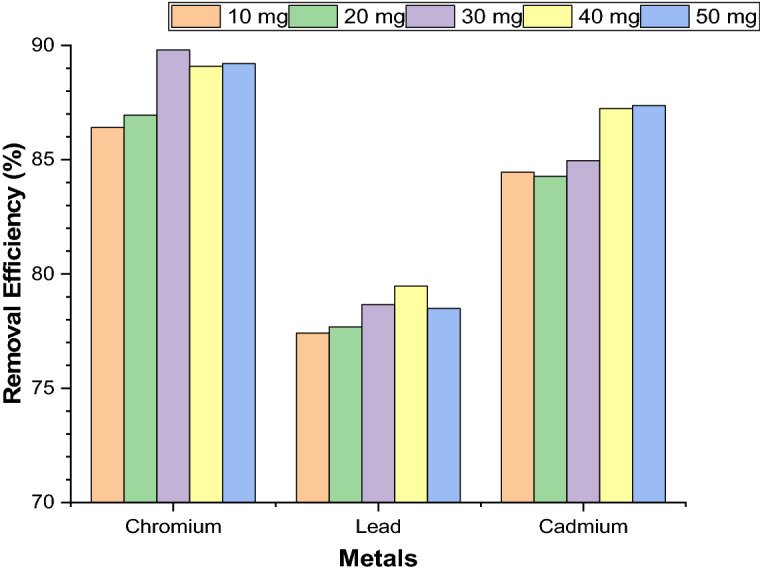
Effect of adsorbent dosage for removal of Cr(VI), Pb(II), and Cd(II).

Adsorbent dosage was varied from 0.01 to 0.05 g, under the specific condition (contact time of 90 min, 200 rpm shaking speed, room temperature and pH of 3 & 5) using Nickel ferrite nanoparticles (NFN’s). The relationship between adsorbent dosage and removal efficiency of metal ions from synthesized wastewater are shown in the Fig. 7.

Nickel ferrite nanoparticles (NFN’s) shown maximum removal efficiency of 89.8% for Cr(VI) ions on 30 mg of adsorbent dosage, 79.47% for Pb(II) ions on 40 mg and 87.24% for Cd(II) ions on 40 mg of adsorbent dosage. The results clearly show that the increased in adsorbent dosage also increases the removal efficiency of metal ions and maximum removal efficiency was attained at a particular adsorbent dosage, until an optimal dosage of 30 mg for Cr (VI), 40 mg for Pb(II) and Cd(II) was reached after which the removal was more or less the same. Starting from 10 mg with an increment of 10 mg, the dosage was increased up to 50 mg showing increase removal with increased dosage until the maximum removal is achieved at the 30 mg which is the optimum dosage for removal of Cr(VI), 40 mg for Pb(II) and Cd(II). Increasing the percentage of adsorption with adsorbent dose is due to the increase in adsorbent surface area and availability of more adsorption sites, the rate of adsorption, however, decreased with increase in adsorbent dose after the optimal value. This is due to overlapping of adsorption sites as a result of overcrowding of adsorbent particles. When the NFN’s were dispersed into synthesized wastewater with high dose of NFN’s it resulted decrease adsorption which was the major cause reduction of contact surface of nanoparticles for removal of metal ions.

### Pseudo-second order kinetic model

The obtained results for the equilibrium concentrations and sorption capacities were compared with the forms of Langmuir and Freundlich isotherms, but did not yield a straight line. The results were then compared against a pseudo-second order kinetic model as done is previous studies for heavy metal removal through nickel ferrite nanoparticles. The expression for the pseudo-second order kinetic model is shown below:

$$\frac{{\mathrm{t}}}{{\mathrm{q}}_{\mathrm{t}}} = \frac{1}{{\mathrm{K}}_{2}{\mathrm{q}}_{\mathrm{e}}^{2}}+ \frac{\mathrm{t}}{{\mathrm{q}}_{\mathrm{e}}}$$

where t (min) is contact time, q_t_ (mg/g) is the sorption capacity at time t, q_e_ (mg/g) is the sorption capacity at equilibrium and K_2_ (g/(mg min)) is the pseudo-second order sorption rate constant^26,29,30^. Sorption capacities at equilibrium and sorption rate coefficients for the adsorption of the three heavy metals through NFN’s along with correlation coefficients are summarized in Table 4.

**Table 4:** Sorption capacities at equilibrium and sorption rate coefficients.

Metal ions	q_e_ calculated (mg/g)	q_e_ experimental (mg/g)	K_2_ (g/mg min)	R^2^
Chromium	21.3025	21.5437	0.0207	0.9995
Lead	19.2150	19.8773	0.0148	0.9996
Cadmium	21.1125	21.5343	0.0198	0.9999

From Table 4, clearly a close agreement among the experimental values and corresponding pseudo-second order kinetic model is observed.

## Conclusion

As a result of this study, low cost spin shaped NFNs using the co-precipitation method were prepared as sorbent for removal of heavy metals. XRD and SEM techniques were used to characterize the synthesized nanostructure materials. XRD analysis indicates that the prepared samples have single phase spinel structure. The SEM image shows that the nanoparticles are spherical, homogeneous and discrete with a particle size around 30–69 nm. These spinel ferrites were found to be efficient for the removal of heavy metals (Cr(VI), Pb(II) & Cd(II)) from synthetic wastewater by adsorption through NFNs magnetic nanoparticles. In this study, the removal efficiencies of Cr(VI), Pb(II) and Cd(II) by using NFNs was determined 89%, 79% and 87% respectively. Optimum dosages of NFNs for the removal of these heavy metals was found to be around 30 mg with generally lower pH values resulting in higher removal efficiencies. The results reiterate the promise of nanoparticles in general and NFNs in particular, for the removal of heavy metals from wastewater. Therefore, spinel ferrite can be considered as a potential candidate in adsorption chemistry for removal of heavy metals from wastewater.
